# Setdb2 Regulates Inflammatory Trigger-Induced Trained Immunity of Macrophages Through Two Different Epigenetic Mechanisms

**DOI:** 10.21203/rs.3.rs-6702384/v1

**Published:** 2025-05-28

**Authors:** Timothy Osborne, Anant Jaiswal, Laszlo Halasz, David Williams

**Affiliations:** Johns Hopkins; Johns Hopkins University; Department of Immunology and Immunotherapy, Icahn School of Medicine, Mount Sinai; Center for Infectious Disease, Inflammation and Immunity, Department of Surgery, East Tennessee State University

**Keywords:** Setdb2, trained immunity, innate immune memory, epigenetic reprogramming, inflammation, β-glucan, western diet feeding, ox-LDL, monocyte/macrophage

## Abstract

“Trained immunity” of innate immune cells occurs through a sequential two-step process where an initial pathogenic or sterile inflammatory trigger is followed by an amplified response to a later un-related secondary pathogen challenge. The memory effect is mediated at least in part through epigenetic modifications of the chromatin landscape. Here, we investigated the role of the epigenetic modifier Setdb2 in microbial (β-glucan) or sterile trigger (Western-diet-WD/oxidized-LDL-oxLDL)-induced trained immunity of macrophages. Using genetic mouse models and genomic analysis, we uncovered a critical role of Setdb2 in regulating proinflammatory and metabolic pathway reprogramming. We further show that Setdb2 regulates trained immunity through two different complementary mechanisms: one where it positively regulates glycolytic and inflammatory pathway genes via enhancer-promoter looping, and is independent of its enzymatic activity; while the second mechanism is associated with both increased promoter associated H3K9 methylation and repression of interferon response pathway genes. Interestingly, while both mechanisms occur in response to pathogenic training, only the chromatin-looping mechanism operates in response to the sterile inflammatory stimulus. These results reveal a previously unknown bifurcation in the downstream pathways that distinguishes between pathogenic and sterile inflammatory signaling responses associated with the innate immune memory response and may provide potential therapeutic opportunities to target cytokine vs. interferon pathways to limit complications of chronic inflammation.

## INTRODUCTION

The vertebrate innate immune system has evolved a robust mechanism that recognizes prior pathogen exposure and directs a rapid and robust secondary response to a broad spectrum of infectious agents to limit deleterious effects on organismal health and survival. This learned response has been described as “trained immunity” or “innate immune memory” and provides protection from a broad range of potential future pathogens.^1^ Specific molecular features associated with disease-causing microbes have been identified that initiate the process, and they range from microbial surface carbohydrates such as β-glucan to engineered mimics including the bacillus Calmette-Guérin (BCG) vaccine.^2,3^ However, endogenous triggers that arise during “sterile” inflammatory challenges, for instance, Western diet (WD) feeding and some intermediary metabolites, can also induce a trained immune response.^4^ The process occurs at least in part through epigenetic reprogramming of chromatin in hematopoietic stem cells, dendritic cells, monocytes, and macrophages linked to genes involved in modulating inflammatory and supporting metabolic pathways.^5,6^

The capacity to mount a second proinflammatory challenge response after the primary exposure inherent to the innate immune memory system are retained over time and can be inherited following cell division, which suggests they are influenced by metastable epigenetic modifications. Consistent with this model, recent reports have revealed the importance of key histone modifications and their modifier enzymes in the mechanism. For instance, mono- or tri-methylation at lysine four of histone H3 (H3K4me1 and H3K4me3) within enhancers and promoters of affected genes contributes to β-glucan-induced trained immunity,^7, 8, 9^ and the response was inhibited in macrophages lacking *Set7*, an enzyme regulating H3K4me1.^10^ Additionally, treatment of cells with pan-methyltransferase inhibitors also resulted in a decrease in the innate immune memory response.^5^ An additional study highlighted the role of the H3K9me2 (di-methylation at lysine-9 position of histone) modifying enzyme G9a as a regulator of trained immunity.^11^ Despite these specific examples, how specific chromatin-dependent modifications influence the training process and whether there is heterogeneity in the downstream secondary responses that are related to the identity or source of the initiating trigger have not been explored.

SET domain bifurcated 2 (Setdb2) is a member of the SET domain-containing KMT1 subfamily of lysine methyltransferases.^12^ The SET domain binds S-adenosyl methionine (SAM), which is a methyl-donating cofactor for virtually all known lysine methyltransferases. KMT1 family members such as SETDB1, G9a, and SUV3-9H1 utilize SAM to methylate histone H3 on lysine 9 (H3K9).^12, 13^ H3K9 methylation is a prominent gene-repressive histone mark, and recent studies have shown Setdb2 is induced early during a proinflammatory response in macrophages and is required for the increase in H3K9me3 that occurs at its target cytokine gene-promoters to decrease their expression and limit runaway inflammation.^14^ Setdb2 was also shown to be involved in regulating H3K9 methylation in diabetic wound-associated macrophages in mice.^15^

In a totally unrelated investigation, we uncovered a different role for Setdb2 in hepatocytes, where we showed that Setdb2 was induced by glucocorticoid receptor (GR) signaling during fasting. We showed that Setdb2 works together with GR to modulate long-range enhancer-promoter interactions in hepatic chromatin to stimulate Insig2 gene expression and repress sterol regulatory element binding protein (SREBP)-dependent lipogenesis during fasting.^16^ Additionally, we found that coactivation of GR target genes by Setdb2 was independent of its putative methyltransferase activity and was associated with a decrease in H3K9 methylation of target gene promoters. In a follow-up study, we also found that Setdb2 was highly expressed in human atherosclerotic lesions, and transplantation of bone marrow from global Setdb2-deficient mice into *Ldlr−/−* mice resulted in increased plaque-associated vascular inflammation with altered immune cell profiles and accelerated atherosclerotic progression. This suggests Setdb2 is involved with chronic inflammation associated with metabolic disorders such as atherosclerosis.^17^

Here, we investigated the role of Setdb2 in response to microbial (β-glucan) or endogenous sterile inflammatory stimuli Western-diet (WD) and oxidized-LDL (oxLDL)) models of the innate immune memory response in macrophages challenged in vivo or in vitro. We compared responses in WT versus two key genetic models: one where Setdb2 is specifically deleted in myeloid cells and another where we knocked in alanine point mutations to alter two key amino acids in the SET domain that are key to coordinate binding to SAM for its methyltransferase activity. The results show that Setdb2 is critical for the induction of trained immunity in macrophages triggered both in vivo and in vitro and in response to pathogenic and sterile inflammatory signals. We combined gene expression analyses with chromatin immunoprecipitation (ChIP) and chromatin conformation capture (3C) analyses to show that Setdb2 plays a central role in regulating proinflammatory and metabolic pathway genes through two different mechanisms. In one, Setdb2 positively regulates glycolytic and inflammatory cytokine pathway genes via changes in enhancer-promoter looping, and this requires Setdb2 but is independent of its enzymatic activity. In the second mechanism, Setdb2 enzyme activity and its SET domain is required to negatively regulate genes involved in inflammatory and interferon pathway signaling, and this is associated with H3K9 methylation changes in target gene promoters. These results reveal a bifurcation of the innate immune memory response into pathways that, on the one hand, activate pro-inflammatory and metabolic responses while inhibiting interferon-associated signaling pathways. They also reveal a divergent memory response initiated by β-glucan vs WD/ox-LDL, which is consistent with different training responses downstream from infectious vs sterile chronic inflammatory triggers.

## RESULTS

### Setdb2 is essential for β-glucan-induced innate immune memory of bone-marrow macrophages

**Figure 1:**
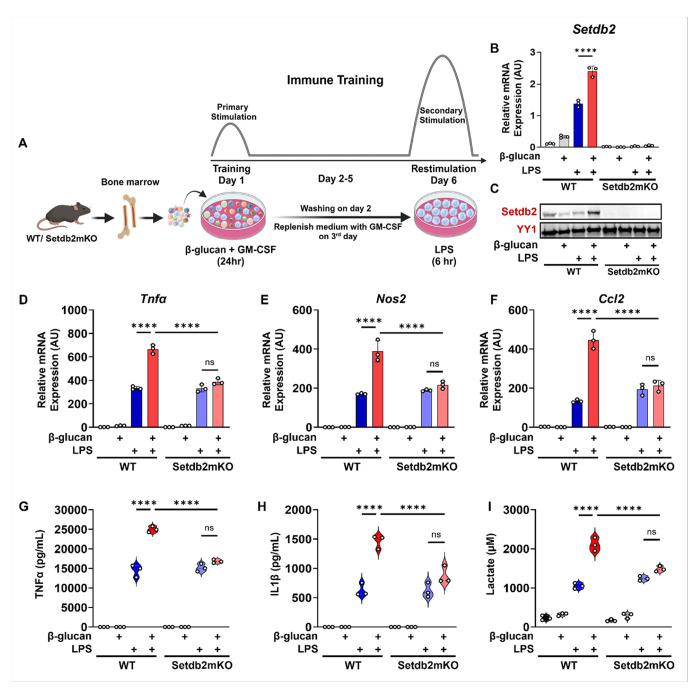
Setdb2 is essential for β-glucan induced innate immune memory of bone-marrow macrophages (A) Graphical outline showing in vitro β-glucan training model. Bone marrow cells were isolated from WT and Setdb2mKO mice and treated with 5 μg/mL β-glucan (training stimulus) or culture medium for 24 h, cultured for 5 days, and restimulated with 100ng/mL LPS (secondary challenge) or culture medium for 6 h on day 6, cells were harvested for analysis. (B) Setdb2 mRNA expression and (C) Setdb2 protein expression, showing one representative immunoblot from three independent experiments. YY1 serves as a loading control, (D) mRNA expression of Tnfα (E) Nos2, and (F) Ccl2, (G) Pro-inflammatory cytokine levels of TNFα, and (H) IL-1/β, and (I) Extracellular lactate levels in untreated control, β-glucan alone, LPS alone, and β-glucan followed by LPS restimulated BM-macrophages from WT (n = 3) and Setdb2mKO mice (n = 3). Data are represented as mean ± SD. ‘n’ represents biological replicates of each strain and treatment type. p-value was calculated using two-way ANOVA with Tukey post-hoc test for multiple comparisons. *p < 0.05, **p < 0.01, and ***p < 0.001, ns, not significant change.

To investigate the role of Setdb2 in innate immune memory, we first established a two-step in vitro culture model of trained immunity in bone marrow (BM)-macrophages exposed to the yeast cell wall carbohydrate β-glucan as a prototype pre-training signal as described by others^10^ and Figure 1A. Briefly, freshly isolated mouse bone marrow cells were incubated with β-glucan or culture medium. After 24 h, cells were washed and cultured with GM-CSF for an additional five days and then treated with culture medium alone or medium supplemented with lipopolysaccharide (LPS) for six hours. The culture supernatant was collected, and cells were harvested for RNA and protein analysis (Figure 1A). Following this protocol, we found that untrained LPS treatment alone induced *Setdb2* mRNA expression. Interestingly, LPS stimulation effect was further amplified in WT cells that were pre-trained with β-glucan in WT (Figure 1B). Setdb2 protein levels followed a similar pattern, and as expected, Setdb2 mRNA and protein expression was negligible in BM-macrophages isolated from mice with a myeloid-specific knockout of Setdb2 (Setdb2mKO) (Figure 1B, C). Thus, the expression of Setdb2 was super-induced by β-glucan training similar to classic trained immunity responses observed in previous reports.^10^

To explore the role of Setdb2 in the training mechanism, we measured expression of mRNAs for key proinflammatory markers of the classic innate immune memory response such as nitric oxide synthase (*Nos2*), tumor necrosis factor (*Tnfα*) and monocyte chemoattractant protein 1 (*Ccl2*). These genes were induced by untrained LPS alone treatment and super-induced by β-glucan pre-training in WT similar to Setdb2 expression as expected. Interestingly, the super-induction by β-glucan pre-training was significantly blunted in BM-macrophages isolated from Setdb2mKO mice (Figure 1D–F). Similarly, secretion of Tnfα and IL1β cytokines from the cells was super-induced in WT, but this was also significantly blunted in Setdb2mKO BM-macrophages (Figure 1G–H). During trained immunity of macrophages, cells also shift their metabolism to increase aerobic glycolysis,^5^ so we also measured extracellular lactate levels as a readout of this metabolic shift. This response was also enhanced by pre-training in WT, and it was significantly reduced in Setdb2mKO BM-macrophages (Figure 1I). Together these findings show that Setdb2 plays a critical role in the innate immune memory response of BM-macrophages.

### Setdb2 regulates key metabolic and inflammatory pathways during β-glucan-induced trained immunity in bone-marrow macrophages

**Figure 2:**
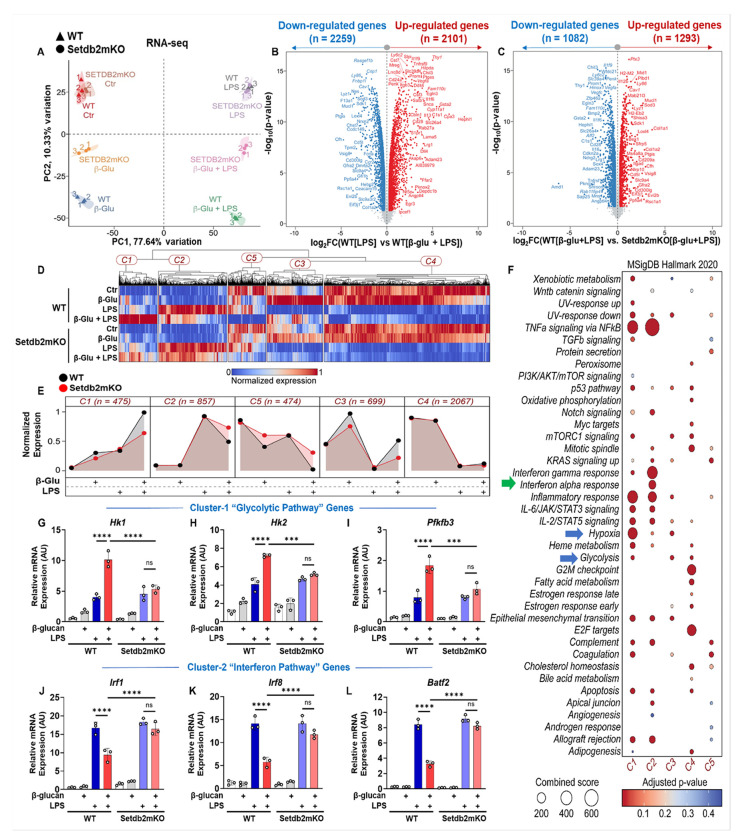
Setdb2 regulates key metabolic and inflammatory pathways during β-glucan-induced trained immunity in bone-marrow macrophages Bone marrow cells were isolated from WT and Setdb2mKO mice and treated with 5 μg/mL β-glucan (training stimulus) or culture medium for 24 h, cultured for 5 days, and restimulated with 100ng/mL LPS (secondary challenge) or culture medium for 6 h on day 6, cells were harvested and analyzed by RNA-sequencing. (A) Principal component analysis (PCA) showing distinct transcriptional profiles across treatment conditions and genotypes (n = 3). (B) Volcano plot visualizing differential gene signatures in cells trained with β-glucan followed by LPS restimulation vs LPS alone treated BM-macrophages from WT (n = 3). (C) Volcano plot visualizing differential gene signatures in cells trained with β-glucan followed by LPS restimulation for WT vs Setdb2mKO BM-macrophages (n = 3). (D) Heatmap, and (E) Area plot showing k-means (k = 5) and hierarchical clustering (n = 3). (“See also Figure S1”) (F) Enrichment analysis using MSigDB to identify significantly enriched biological processes associated with the DEGs across experimental groups (n = 3). (G) mRNA expression of Hk1 (H) Hk2, (I) Pfkfb3, (J) Irf1, (K) Irf8, and (L) Batf2 in untreated control, β-glucan alone, LPS alone, and β-glucan followed by LPS restimulated BM-macrophages from WT (n = 3) and Setdb2mKO mice (n = 3). Statistical analysis for RNA-seq data was performed using DESeq2 with its default settings. Data are represented as mean ± SD. ‘n’ represents biological replicates of each strain and treatment type. p-value was calculated using two-way ANOVA with Tukey post-hoc test for multiple comparisons. *p < 0.05, **p < 0.01, and ***p < 0.001, ns, not significant change.

To explore the effects of Setdb2 on the global transcriptional program during the trained immune response, we performed whole-genome RNA sequencing of β-glucan-pre-trained WT vs. Setdb2mKO BM-macrophages. Principal component analysis revealed that control cells from WT and Setdb2mKO mice clustered together, which is consistent with Setdb2 not having a dominant role in unstimulated cells. However, the PCA patterns were shifted for all treatment groups, and there were clear differing patterns for cells from WT vs. Setdb2mKO, consistent with a key role of Setdb2 in the training process (Figure 2A). Over 2000 genes were differentially regulated comparing β-glucan pre-trained versus untrained LPS-treated BM-macrophages, as shown by the volcano plots in Figure 2B and more detailed in supplemental table S2. More than 1000 of these were differentially expressed in β-glucan pre-trained cells followed by challenged with LPS from WT vs Setdb2mKO mice (Figure 2C).

Unsupervised k-means clustering revealed a broad pattern of transcriptional changes that were distributed into 5 unique clusters (k=5) visualized by the heatmaps in Figure 2D and Figure S1A-E. The cluster effects were pooled together to generate a cluster-wide response using a line plot format that emphasizes the dynamic nature of the transcriptional profiles for the five clusters. Importantly, the expression pattern for cluster-1 (475 genes) and cluster-2 (857 genes) displayed interesting transcriptional profiles with respect to Setdb2 loss and β-glucan pre-training. Genes associated with cluster-1 were induced by LPS treatment alone and super-induced by β-glucan pre-training followed by LPS stimulation in WT, while this super-induction was significantly blunted in Setdb2-deficient BM-macrophages. In contrast, cluster-2 associated genes were induced by LPS, but the LPS-mediated induction was inhibited by β-glucan pre-training in WT. However, this inhibition by β-glucan pre-training was lost in the Setdb2mKO BM-macrophages.

To explore the biological processes and molecular pathways associated with the transcriptional profiles, we performed a gene enrichment analysis using Molecular Signatures Database (MSigDB) on the genes contained in all five clusters (Figure 2F). This analysis showed that the majority of genes from cluster-1 and cluster-2 were associated with proinflammatory and metabolic pathways in comparison with other clusters. Cluster-1 genes were specifically enriched in proinflammatory and metabolic pathways including hypoxia and glycolysis, while cluster-2 genes were preferentially associated with interferon alpha and gamma pathways. Some proinflammatory pathways, such as JAK/STAT, as well as TNFα signaling via *NFκB*, were equally distributed within both cluster-1 and 2 enriched genes. Genes in the other clusters were associated with other processes, and because their overall response to treatment and genotype was more complex, we focused on clusters 1 and 2.

To validate our RNA-sequencing data, we performed qPCR analysis to measure key genes in clusters 1 and 2. Consistent with the RNA-seq data, we found that cluster-1-associated glycolytic genes, *i.e., Hk1, Hk2, and Pfkfb3*, were significantly induced by LPS and super-induced by β-glucan pre-training in WT, similar to *Tnfα, Nos2, and Ccl2*, and this response was blunted in Setdb2mKO BM-macrophages (Figure 2G–I). Similarly, consistent with the RNA-seq results, cluster-2-associated interferon pathway genes, *i.e., Irf1, Irf8, and Batf2*, were induced by LPS but this was inhibited by β-glucan pre-training in WT, but the inhibition was also lost in Setdb2mKO BM macrophages (Figure 2J–L). Taken together, these results confirm that Setdb2 is required for both β-glucan training-dependent activation of cluster-1 genes and β-glucan training-dependent suppression of cluster-2 genes.

### Setdb2 regulates in vivo β-glucan induced trained immunity in peritoneal macrophages

**Figure 3:**
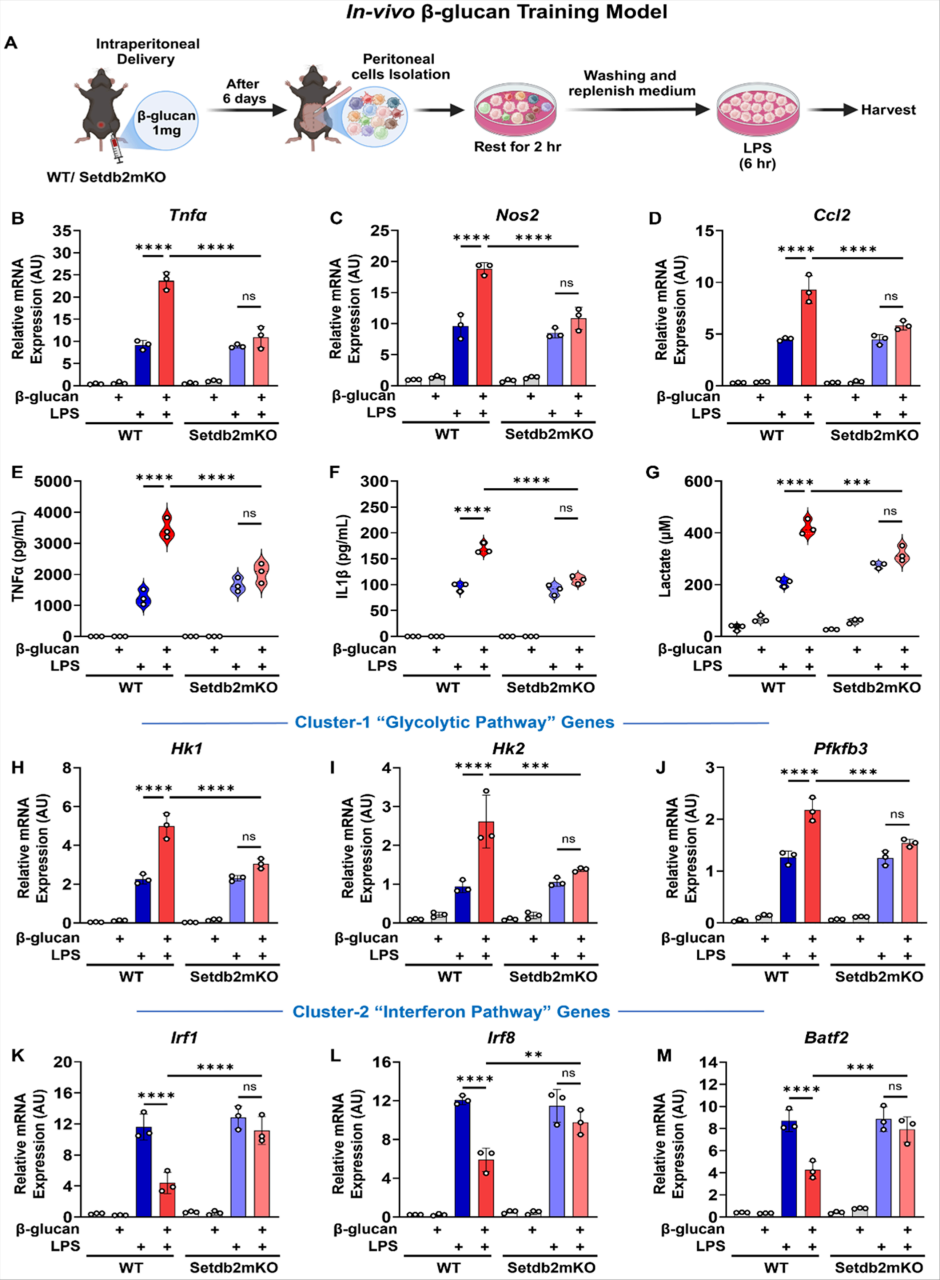
Setdb2 regulates in-vivo β-glucan induced trained immunity in peritoneal macrophages (A) Graphical outline showing in vivo β-glucan training model. WT or Setdb2mKO mice were peritoneally injected with 1mg β-glucan (training stimulus) or PBS alone and after 5 days peritoneal macrophages were isolated and restimulated with 100ng/mL LPS (secondary challenge) or culture medium for 6 h. Cells were harvested for analysis. (B) mRNA expression of Tnfα, (C) Nos2, and (D) Ccl2, (E) Pro-inflammatory cytokine levels of TNFα, and (F) IL-1β, (G) Extracellular lactate levels, (H) mRNA expression of Hk1 (I) Hk2, (J) Pfkfb3, (K) Irf1 (L) Irf8, and (M) Batf2 in untreated control, β-glucan alone, LPS alone, and β-glucan followed by LPS restimulated peritoneal macrophages from WT (n = 3) or Setdb2mKO mice (n = 3). Data are represented as mean ± SD. ‘n’ represents biological replicates of each strain and treatment type. p-value was calculated using two-way ANOVA with Tukey post-hoc test for multiple comparisons. *p < 0.05, **p < 0.01, and ***p < 0.001, ns, not significant change.

To validate our *in vitro* findings further and to analyze the effect of β-glucan pre-training *in vivo*, β-glucan was injected into the peritoneal cavity of WT and Setdb2mKO mice. After 6 days we isolated the peritoneal macrophages and treated them acutely with LPS as a secondary stimulus (diagrammed in Figure 3A). Similar to the β-glucan in-vitro challenge, we found that expression of proinflammatory genes in cluster-1 including *Tnfα, Nos2*, and *Ccl2* and secretion of Tnfα and IL-1β cytokines were super-induced by β-glucan training in vivo, and these responses were blunted in Setdb2mKO peritoneal macrophages (Figure 3B–F). There was a similar effect on lactate production and the expression of cluster-1 associated glycolytic genes, i.e., *Hk1, Hk2*, and *Pfkfb3* (Figure 3G–J). In contrast, but similar to our in vitro training studies, the LPS induction of cluster-2 interferon pathway genes (i.e., *Irf1, Irf8, Batf2*) was blunted by β-glucan pre-training in WT, and this was not observed in Setdb2mKO (Figure 3K–M). Overall, our data provide both in vitro and in vivo evidence that Setdb2 regulates β-glucan training-mediated induction and repression of cluster 1 and 2 genes, respectively.

### β-glucan-dependent trained immunity in BM-macrophages from Setdb2 knock-in mice

**Figure 4:**
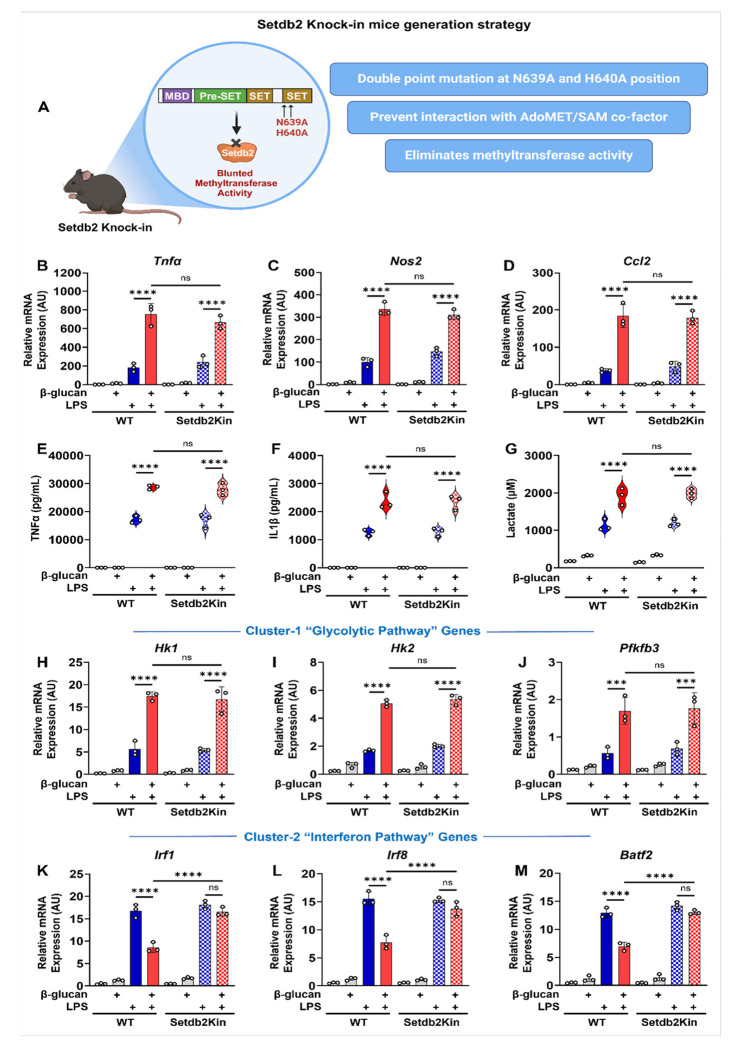
β-glucan-dependent trained immunity in BM-macrophages from WT vs. Setdb2 knock-in mice (A) Schematic diagram showing Setdb2 knock-in strategy (“See also Figure S2”). Bone marrow cells from WT and Setdb2Kin mice were treated with 5 μg/mL β-glucan (training stimulus) or culture medium for 24 h, cultured for 5 days, and restimulated with 100ng/mL LPS (secondary challenge) or culture medium for 6 h on day 6. Cells were harvested for analysis. (B) mRNA expression of Tnfα, (C) Nos2, and (D) Ccl2, (E) Pro-inflammatory cytokines TNFα, and (F) IL-1β levels, (G) Extracellular lactate levels, (H) mRNA expression of Hk1 (I) Hk2, (J) Pfkfb3, (K) Irf1 (L) Irf8, and (M) Batf2 in untreated control, β-glucan alone, LPS alone, and β-glucan followed by LPS restimulated BM-macrophages from WT (n = 3) and Setdb2Kin mice (n = 3). Data are represented as mean ± SD. ‘n’ represents biological replicates of each strain and treatment type. p-value was calculated using two-way ANOVA with Tukey post-hoc test for multiple comparisons. *p < 0.05, **p < 0.01, and ***p < 0.001, ns, not significant change.

Setdb2 is a member of the KMT1 family of lysine methyltransferases, which includes several well-characterized enzymes that methylate lysine 9 on histone 3 to repress transcription.^13^ Consistent with this notion, recent studies have suggested that Setdb2 is linked with gene repression, where it is associated with an increase in H3K9me3 levels in the promoters of genes that are proposed to be suppressed by Setdb2 during proinflammatory stimulation in macrophages.^14,15^ However, the differential effect of Setdb2 on the response patterns for cluster-1 vs. cluster-2 genes in the current study suggests Setdb2 may regulate cluster-1 positively while inhibiting expression of cluster-2 genes in response to β-glucan training. To explore whether Setdb2’s methyltransferase activity might be involved in differential regulation of cluster-1 vs. cluster-2 pathway genes, we developed a knock-in mouse line (Setdb2Kin), which has germline mutations that change two critical amino acids of the conserved SET domain to alanine. Based on the structure of the SET domain bound to SAM,^12^ these mutations will prevent binding of this required cofactor and eliminate any direct methyltransferase activity (Figure 4A). We found the knock-in mice were viable, fertile, and showed no overt signs of lethality when grown under standard laboratory conditions. Sanger sequencing further confirmed the two-point mutations were introduced into the SET domain of the Setdb2 gene in Setdb2Kin mice (Figure S2A-C). Next, we compared BM-macrophages from the Setdb2Kin to WT mice in the β-glucan training protocol. Interestingly, unlike in Setdb2mKO, we found that the LPS-dependent induction of mRNAs encoding cluster-1 proinflammatory genes in Setdb2Kin such as *Tnfα, Nos2*, and *Ccl2* were still super induced by β-glucan pre-training similar to WT (Figure 4B–D). Similar results were observed for the secretion of TNFα, IL-1β, and lactate levels (Figure 4E–G). Cluster-1 associated glycolytic pathway genes *Hk1, Hk2*, and *Pfkfb3* were also super-induced by β-glucan pre-training in the Setdb2Kin similar to WT (Figure 4H–J). In contrast, the β-glucan dependent repression of the LPS-induction of cluster-2-associated interferon pathway genes, *Irf1, Irf8*, and *Batf2*, was blocked in Setdb2Kin BM-macrophages (Figure 4K–M), which was similar to results for Setdb2mKO. Taken together, these results suggest that Setdb2 may have different roles in regulating cluster-1 and cluster-2 associated pathways.

### Setdb2 regulates innate immune memory via two distinct mechanisms: activation through chromatin looping and suppression through its methyltransferase activity

Our lab previously reported that Setdb2 is induced during fasting in the liver, where it functions together with the glucocorticoid receptor (GR) to activate a subset of GR target genes involved in the metabolic adaptation to fasting. In this role, Setdb2 was associated with long-range enhancer-promoter interactions and was independent of its enzymatic activity.^16^ Thus, based on these prior findings and considering the results presented above for Setdb2mKO vs. Setdb2Kin macrophages, we hypothesized that Setdb2 may regulate cluster-1 gene activation through enhancer-promoter looping while repressing cluster-2 genes through its methyltransferase activity.

**Figure 5:**
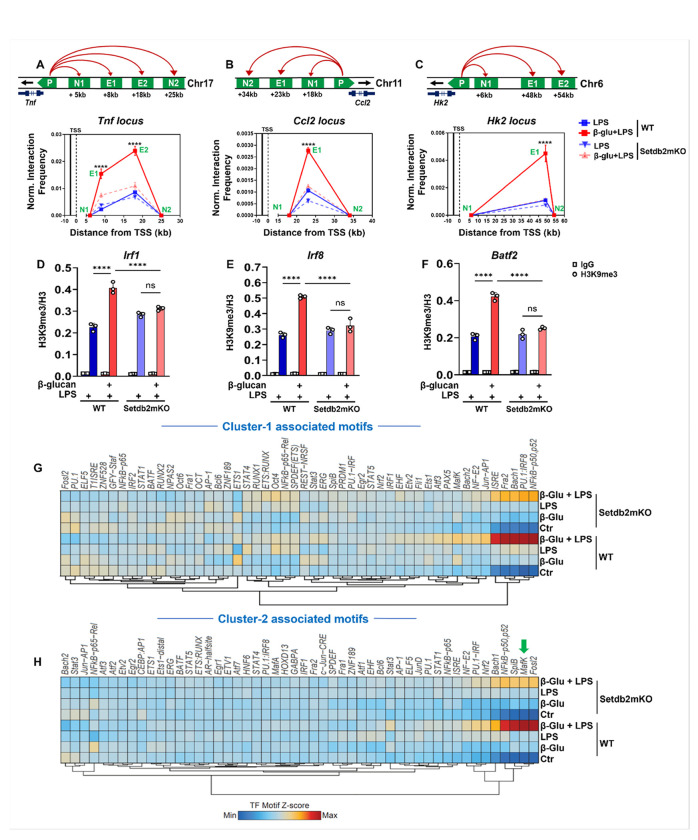
Setdb2 regulates innate immune memory via two distinct mechanisms: activation through chromatin looping and suppression through its methyltransferase activity Bone marrow cells were isolated from WT and Setdb2mKO mice and treated with 5 μg/mL β-glucan (training stimulus) or culture medium for 24 h, cultured for 5 days, and restimulated with 100ng/mL LPS (secondary challenge) or culture medium for 6 h on day 6. Chromatin was harvested and analyzed by 3C-qPCR, ChIP-qPCR and ATACseq analysis. (A) Diagram representing the chromosomal loci at respective genes, P=promoter region, N= negative bait, E= enhancer bait for potential long-range enhancer-promoter interaction through 3C-qPCR analysis at Tnfα, (B) Ccl2, or (C) Hk2 locus, enhancer bait regions for 3C-qPCR spans the promoter regions from TSS to +8 kb and +18 kb for Tnfα, +23kb for Ccl2, and +48kb regions for Hk2 loci. (D) H3K9me3 levels (ChIP-qPCR) at Irf1 (E) Irf8, and (F) Batf2 promoter in chromatin of LPS alone, or β-glucan followed by LPS restimulated BM-macrophages from WT (n = 3) or Setdb2mKO mice (n = 3). (“See also Figure S3”) (G) ChromVAR z-scores heatmap representing significantly enriched HOMER motifs for differentially opened chromatin regions in cluster 1 and (H) cluster-2 gene loci from ATAC-seq data performed with chromatin from untreated control, β-glucan alone, LPS alone, and β-glucan followed by LPS restimulated BM-macrophages from WT (n = 3) and Setdb2mKO mice (n = 3). (“See also Figure S4”) For ATAC-seq data, differential peak accessibility was analyzed using DiffBind with EdgeR, employing default parameters. Data are represented as mean ± SD. ‘n’ represents biological replicates of each strain and treatment type. p-value was calculated using two-way ANOVA with Tukey post-hoc test for multiple comparisons. *p < 0.05, **p < 0.01, and ***p < 0.001, ns, not significant change.

To test our hypothesis, we first performed chromosome conformation capture (3C-qPCR) to analyze long-range enhancer-promoter interactions adjacent to cluster-1 genes. We targeted the promoter and enhancers for *Tnfα, Ccl2*, and *Hk2*, all cluster-1 genes, in chromatin isolated from BM-macrophages subjected to the in vitro β-glucan training protocol. Our 3C-qPCR data demonstrated a β-glucan pre-training dependent increase in the association between the +8 kb and +18 kb enhancer regions for *Tnfα*, a +23 kb for *Ccl2*, and a +48 kb upstream enhancer region for *Hk2*, with the corresponding transcription start sites (TSS) for each gene in chromatin from WT vs Setdb2mKO BM-macrophages. The data show significant enhancer-promoter interactions that were more robust in chromatin isolated from LPS-treated BM-macrophages that were pre-trained with β-glucan compared to chromatin from untrained samples treated only with LPS. Importantly, this response was significantly lower in chromatin from Setdb2mKO mice BM-macrophages (Figure 5A–C). These results suggest that Setdb2 acts to facilitate enhancer-promoter interactions that influence cluster-1 pathway genes during the β-glucan training response.

Next, to determine whether Setdb2 suppression of cluster-2 genes might be mediated through changes in promoter-associated H3K9 methylation, we performed ChIP-qPCR to measure H3K9 methylation levels at the promoters of cluster-2-specific interferon pathway genes. H3K9 tri-methylation levels were significantly increased by β-glucan pre-training at the promoters of *Irf1, Irf8*, and *Batf2* genes in WT mice, as compared to untrained samples treated only with LPS (Figure 5D–F). Interestingly, H3K9 promoter methylation levels were also significantly decreased in Setdb2mKO BM-macrophages. We also evaluated H3K9 mono (Figure S3A-C) and di-methylation (Figure S3D-F) levels and these chromatin marks also followed the same pattern. In contrast, there were no major changes observed with total histone H3 levels at these promoter sites (Figure S3G-I). Overall, these data suggest that cluster-2 pathway gene repression by β-glucan pre-training was associated with elevated levels of promoter-specific H3K9 methylation, which is consistent with a role for Setdb2’s catalytic activity being required for the reduced levels of gene expression. (Figures 5D–F)

To interrogate the chromatin accessibility landscape that is influenced by β-glucan training, we performed Assay for Transposase-Accessible Chromatin sequencing (ATAC-seq) and analyzed the peak data using ChromVar to gain insights into the potential motifs that are associated with differential peaks across the different treatment groups. This analysis showed significant differences in peak distribution comparing β-glucan-trained LPS-challenged WT vs. Setdb2mKO BM-macrophages. A detailed analysis of the motifs associated with variation between the two genotypes showed (Figure S4 B, C) that *NFκB* motif was highly enriched within open chromatin regions associated with both cluster 1 and 2 pathway genes in WT chromatin and the signal was significantly lower in the Setdb2mKO samples (Figure S4A and Figure 5 G–H). This is consistent with our RNA seq data, where *NFκB* signaling genes are present in both clusters. The comparison also showed that the repressor *MafK* motif was enriched within the cluster-2 specific peaks (Figure 5I) which is interesting because MafK has been shown to be recruited along with Setdb1 and the SAM-synthesizing MatIIa/MatIIb dimer to repress target gene expression.^18^

### Setdb2 regulates ‘western-diet-induced’ innate immune training of bone-marrow monocytes

In a previous study, we reported that an adoptive transfer of bone marrow from mice with a global deletion of Setdb2 into irradiated *Ldlr*-knockout mice resulted in increased vascular inflammation and elevated progression of atherosclerosis when the mice were fed a western diet (WD) relative to mice that were given bone marrow from WT mice.^17^ Recent literature also indicates that initial exposure of innate immune cells to endogenous atherogenic stimuli during WD feeding in mice results in long-lasting hyperresponsive immune cells in the bone marrow. This “training” effect in bone marrow cells leads to functional reprogramming, which eventually worsens atherosclerosis.^4^

**Figure 6:**
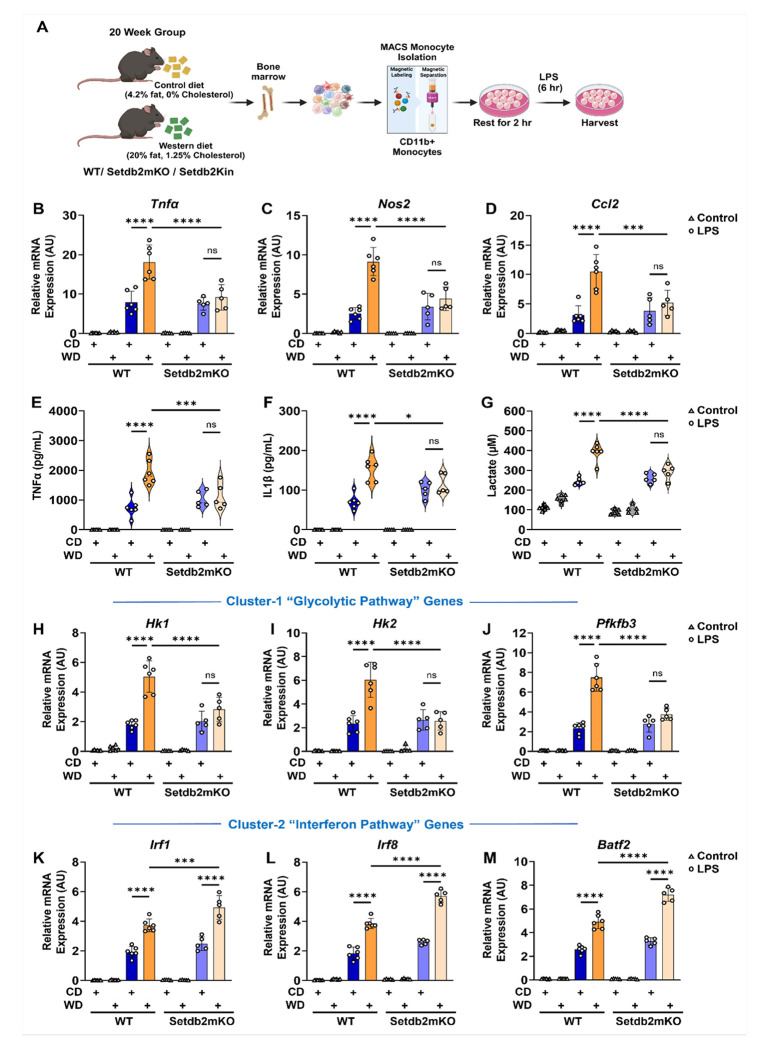
Setdb2 regulates ‘western-diet induced’ innate immune memory of bone-marrow monocytes (A) Graphical outline showing in vivo western diet-induced training model. CD11b^+^ BM-monocytes were isolated from control-diet or western-diet fed WT or Setdb2mKO mice followed by stimulation with 100ng/mL LPS or culture medium in vitro for 6 h. (“See also Figure S5”) (B) mRNA expression of Tnfα (C) Nos2, and (D) Ccl2, (E) Pro-inflammatory cytokine levels of TNFα, and (F) IL-1β, (G) Extracellular lactate levels, (H) mRNA expression of Hk1 (I) Hk2, (J) Pfkfb3 (K) Irf1 (L) Irf8, and (M) Batf2 in control diet, western diet, control diet followed by in vitro LPS restimulation, and western diet followed by in vitro LPS restimulated BM-monocytes isolated from WT and Setdb2mKO mice. Control diet WT (n=6), Control diet Setdb2mKO (n=5), Western diet WT (n=6), and Western diet Setdb2mKO mice (n=5) from one of the three independent experimental group. Data are represented as mean ± SD. ‘n’ represents biological replicates of each strain and treatment type. p-value was calculated using two-way ANOVA with Tukey post-hoc test for multiple comparisons. *p < 0.05, **p < 0.01, and ***p < 0.001, ns, not significant change.

To determine whether Setdb2 might also be involved in the early reprogramming of bone marrow cell during WD-induced immune training, we fed WT, Setdb2mKO and Setdb2Kin mice with a WD for 20 weeks. This resulted in similar increases in body weight, liver weight, and serum cholesterol levels in all three genotypes (Figure S5A-F). Next, to evaluate whether the loss of Setdb2 affected the pattern of innate immune training in WD-fed mice, we isolated CD11b+ monocytes from the bone marrow of control and WD-fed WT and Setdb2mKO mice and challenged them acutely with LPS (Figure 6A). qPCR analysis for cluster-1 associated pathway genes showed that the WD feeding resulted in a significantly more robust response to LPS challenge compared to control diet-fed mice, consistent with an in vivo training effect in WD-fed WT mice (Figure 6B–D). However, similar to the results for β-glucan training, this enhanced response was not observed in Setdb2mKO monocytes. The expression of key cluster 1-specific glycolytic genes and the secretion of cytokines TNFα and IL1β and lactate were all similarly affected (Figure 6E–J). Consistent with the β-glucan training results, the WD-dependent super-induction following LPS stimulation was dramatically blunted in monocytes from Setdb2mKO mice. Interestingly, in contrast to our observations where β-glucan pre-training resulted in suppression of the LPS activation of cluster-2 specific interferon pathway genes, WD feeding resulted in significant induction of these genes by LPS in both WT and Setdb2mKO samples. In fact, the induction of cluster 2 genes was even more robust in WD-fed Setdb2mKO monocytes (Figure 6K–M).

## DISCUSSION

In this study, we identified a dual role for Setdb2 in the innate immune memory response of monocytes/macrophages. When bone marrow-derived cells were treated with either β-glucan or oxidized LDL in vitro as a training agent and then cultured for several days followed by secondary stimulation with LPS on day 6, the cells pre-treated with either training compound displayed a more robust secretion of TNF and IL-1b cytokine in response to the secondary exposure to LPS relative to cells treated only with LPS. There was also enhanced secretion of lactate consistent with the metabolic switch to aerobic glycolysis. Importantly, these responses were accompanied by parallel changes in expression of the corresponding cytokine and glycolytic genes consistent with a transcriptional regulatory mechanism of action. These responses to the two-step training protocol represent the classic measures for the innate immune memory response reported over a decade ago by *Netea and colleagues*.^1,2^ Subsequent studies have demonstrated that the initial stimulus leads in part to chromatin-dependent reprogramming that is established following the training exposure, which can be retained over the course of several days in culture. The reprogramming provides a poised platform to support the more robust response to secondary exposure to LPS. We showed that the training response to LPS was significantly blunted in cells isolated from Setdb2mKO mice. RNA-seq comparisons between WT and Setdb2mKO macrophages revealed 5 unique clusters of gene expression response patterns. Cluster 1 genes were super-induced by LPS in response to β-glucan pre-training, whereas the LPS-mediated induction of cluster 2 genes on day 6 was blunted by β-glucan pre-exposure. Interestingly, both responses were lost in cells from Setdb2mKO mice, consistent with a role for Setdb2 in β-glucan pretraining of genes in both clusters. This differential response uncovered a dual role for Setdb2: stimulating expression of cluster 1 genes while suppressing expression of cluster 2 genes, respectively.

Setdb2 is a member of the KMT1 family of lysine methyltransferases, several of which are known to methylate lysine 9 of histone H3, a modification long known to be associated with inhibition of gene expression. Consistent with this role, others have shown Setdb2 is induced by interferon signaling during the early proinflammatory response in macrophages where it binds to promoters encoding key proinflammatory genes, which are normally silenced later to prevent runaway inflammation. In this context, Setdb2 binding is associated with increased methylation of H3K9, and loss of Setdb2 leads to prolonged stimulation of its target proinflammatory genes coincident with the decrease in promoter-associated lysine 9 methylation. This suggests Setdb2 represses inflammatory gene expression through its lysine methyltransferase function.

In another study, our lab showed that Setdb2 is induced by fasting in the liver, where it interacts with GR to co-activate a subset of GR target genes involved in the hepatic fasting response. In this earlier report, we showed Setdb2 is associated with increased enhancer-promoter looping, gene activation, and reduced H3K9 promoter methylation. Additionally, we showed that a mutant version of Setdb2 that altered key residues involved in binding the methyl-donating SAM cofactor still functioned normally to co-activate GR target genes. These results are consistent with a role for Setdb2 in gene activation that is independent of its potential enzyme activity.

To investigate the catalytic and non-catalytic roles for Setdb2 in the innate training response, we engineered a knock-in mouse model (Setdb2Kin) where two critical amino acids in the SET domain that are critical for SAM binding were changed to alanines. These substitutions were designed to prevent interaction with SAM based on the known structure of the SET domain,^12^ which would eliminate any potential inherent methylation activity. When we repeated the β-glucan training study with Setdb2Kin bone marrow-derived cells, the response to β-glucan training for the super-induction of cluster 1 genes by LPS was maintained, whereas the negative effect on LPS activation of cluster 2 genes was lost. When taken together with the loss of both responses in the Setdb2mKO mouse, these results are consistent with a dual role for Setdb2 in β-glucan-dependent training: a positive role in activation of cluster 1 and a negative role in cluster 2 gene responses.

Based on these two opposite responses and the previous results from our lab and others, we hypothesized that enhancer promoter looping was involved in Setdb2 regulation of cluster 1 gene activation, whereas H3K9 methylation was involved in repression of cluster 2 gene expression. To test these predictions, we utilized 3C to assess enhancer-promoter looping in cluster 1 genes, and we utilized direct ChIP analyses for H3K9 methylation to interrogate cluster 2 gene responses during the two-step β-glucan training experiment. The results showed that cluster 1 and 2 genes were associated with enhancer-promoter looping and H3K9 methylation changes, respectively, consistent with our hypothesis.

This dual role for Setdb2 is similar to the actions of other chromatin modifiers, including G9a and JMJD1A. Similar to Setdb2, G9a is also a member of the KMT1 family of lysine methyltransferases and generates H3K9me2 at target genes to effect gene silencing. However, it also acts as a molecular scaffold factor in the assembly of a larger complex with other coactivators during gene activation where its enzyme activity is not required.^21^ Similarly, JMJD1a is an H3K9 demethylase, and its demethylase activity is involved in the stimulation of white adipose “browning,” but other studies also showed it is involved in enhancer-promoter looping to activate a set of thermogenic target genes in brown adipose tissue, and introducing mutations that eliminate its demethylase activity do not affect its role in chromatin looping.^22^

We also performed ATAC-seq analysis on β-glucan-trained WT vs. Setdb2mKO following the secondary LPS challenge. These studies identified enriched motifs within open chromatin regions associated with the promoters of genes in clusters 1 and 2. As expected, the *NFκB* motif was significantly associated with changing peaks proximal to genes for both clusters. However, the *mafK* motif was only highly associated with peaks close to cluster 2 genes. This is interesting because *mafK* is co-recruited along with the SAM synthesizing *matIIa/b* enzyme complex to chromatin to provide the methyl donor needed for H3K9 methylation in concert with Setdb1.^18^Interestingly, Setdb1 is the only other SET enzyme within the KMT1 family with a bifurcated SET domain.^13^ Further studies are required to determine whether Setdb2 might also be recruited to suppress cluster 2 gene promoters through mafK or a related repressor.

Metabolic disorders, including atherosclerosis, are associated with chronic low-grade inflammation, where the innate immune system contributes to vascular inflammation associated with atherosclerotic plaque formation.^23^ Monocyte infiltration into the arterial wall followed by local responses including hypoxia and accumulation of excess modified lipids results in foam cell formation which accelerates atherosclerosis progression. In fact, the inhibition of monocyte infiltration has protective effects in experimental mouse models of atherosclerosis.^24^ However, it is important to note that the appearance of inflammatory monocytes is not only found within plaques but also in the circulation.^25, 26^ In this regard, a recent study in mice also showed that WD feeding leads to reprogramming of the myeloid cell compartment within the bone marrow, leading to a chronic low-grade state of “sterile inflammation”. It also leads to long-lasting reprogramming of circulating myeloid cells, resulting in a hyper-response to a wide range of different secondary immune stimuli.^4^ These studies suggest that ‘sterile inflammatory’ conditions also induce an innate immune memory response similar to infectious triggers like β-glucan.

In a previous study, we reported that bone marrow transplantation from global Setdb2 deficient mice into WD fed *Ldlr*−/− mice resulted in increased vascular inflammation and aggravated atherosclerosis relative to parallel transplants using bone marrow from control WT mice. In the current study we showed that monocytes isolated from WD-fed WT mice were hyper-responsive to secondary LPS challenge in vitro. We also showed that β-glucan injection into mice also resulted in a similar training response to LPS in macrophages challenged with LPS in vitro. These results are similar to prior reports and are consistent with WD feeding and β-glucan providing an initial signal that effectively marks circulating monocytes similar to the adaptation effect of β-glucan exposure in vitro.

The training response in macrophages isolated from mice after β-glucan injection was similar to the response observed with bone marrow cells treated with β-glucan in vitro, where the induction and repression of cluster 1 and 2 genes was observed, and both responses were lost in the Setdb2mKO mice. However, the results when WD feeding was the source of the training signal were different. The super-induction of cluster 1 genes in response to LPS challenge was abolished in cells from Setdb2mKO, but the inhibition of the LPS response for cluster 2 genes was not observed. In fact, if anything, cluster 2 genes were induced even more robustly by LPS challenge than in cells from WT mice. WD feeding is associated with metabolic syndrome and chronic inflammation which is influenced by several intrinsic factors that accumulate throughout this multiweek feeding regimen. One of the key factors generated during WD feeding is Oxidized LDL (oxLDL), which has been suggested to be a key driver of the chronic inflammatory responses associated with metabolic diseases including atherosclerosis.^19^ In fact, studies showed that in vitro challenge of bone marrow-derived cells with oxLDL elicited a similar training response to β-glucan consistent with oxLDL functioning as a major driver of WD associated chronic inflammatory responses. When we repeated the in vitro training experiment using oxLDL to challenge WT and Setdb2mKO cells, we observed a very similar response to the in vivo WD training protocol. The super-activation of cluster 1 genes was lost, but the cluster 2 repression response observed in trained WT cells was not observed. In fact, similar to WD training in vivo, cluster 2 genes were stimulated even more robustly by LPS challenge in the oxLDL-pretrained Setdb2mKO cells. Taken together, these results demonstrate that the loss of Setdb2 leads to a bifurcation in the responses elicited by the two different training agents.

Using single-cell RNA-seq on CD45+ cells in the atherosclerosis studies mentioned above, we found that the aggravated atherosclerotic plaques from the mice reconstituted with Setdb2-deficient bone marrow contained significantly fewer T-cells along with significantly enhanced neutrophil infiltration.^17^ A more detailed analysis of our bulk RNAseq data from the β-glucan training experiment reported here showed an enrichment for Ccl chemokine signaling mediators within cluster-1 genes, whereas Cxcl pathway regulators were enriched in cluster 2. Because cluster 1 and 2 genes are uniquely under- and over-represented in the Setdb2mKO, this analysis also reveals a potential underlying mechanism for the differential appearance of T-cell and neutrophil populations in Setdb2-deficient atherosclerotic plaques.

Overall, our evaluation of Setdb2 in the innate immune training response has uncovered an essential role for Setdb2 that involves a previously unknown branching in the downstream pathways from the initial trigger that define the memory response. Our study also reveals that Setdb2 plays a key role in different response patterns to infectious (β-glucan) vs. sterile (WD/oxLDL) inflammatory triggers. Thus, our results not only provide evidence for significant differences in the training response to two different major pro-inflammatory activation pathways, but they also reveal key mechanistic insight into Setdb2’s mode of action for differential transcriptional regulation of the key innate immune response pathways that are affected. This opens up the possibility that targeting Setdb2’s enzymatic vs. scaffold activity may result in context-dependent potential therapeutic targeting of the divergent pathways in inflammatory or metabolic disorders.

## Figures and Tables

**Figure 7:**
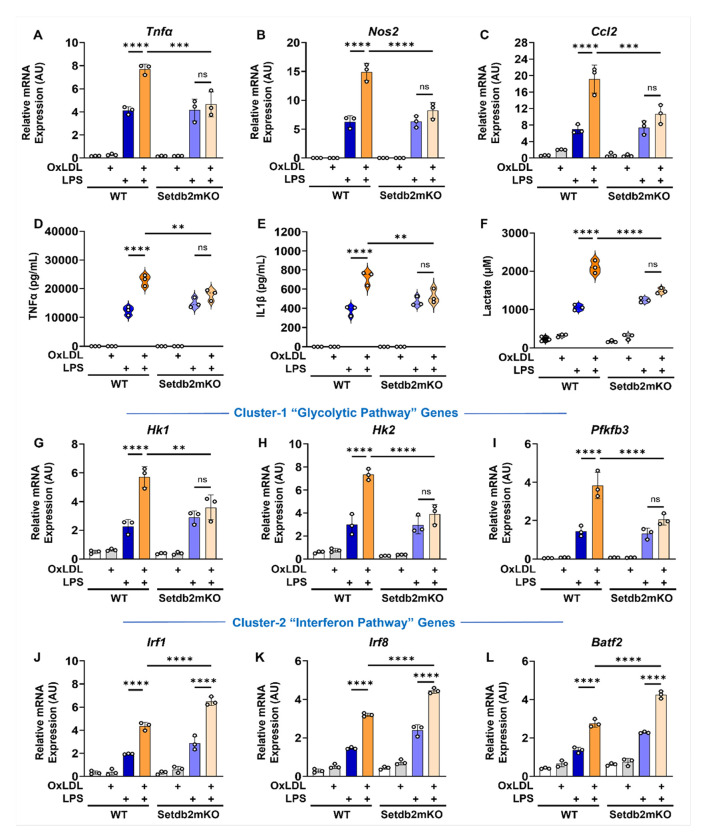
In-vitro training with oxidized-LDL regulates trained immunity responses in bone marrow macrophages similar to ‘Western-diet’ feeding Bone marrow cells from WT and Setdb2mKO mice were isolated and treated with 10 μg/mL oxLDL (training stimulus) or culture medium for 24 h, cultured for 5 days, and restimulated with 100ng/mL LPS (secondary challenge) or culture medium for 6 h on day 6. Cells were harvested for analysis. (A) mRNA expression of Tnfα, (B) Nos2, and (C) Ccl2, (D) Pro-inflammatory cytokine levels of TNFα, and (E) IL-1β, (F) Extracellular lactate levels, (G) mRNA expression of Hk1 (H) Hk2, (I) Pfkfb3, (J) Irf1 (K) Irf8, and (L) Batf2 in untreated control, oxLDL alone, LPS alone, and oxLDL followed by LPS restimulated BM-macrophages from WT (n = 3) or Setdb2mKO mice (n = 3). Data are represented as mean ± SD. ‘n’ represents biological replicates of each strain and treatment type. p-value was calculated using two-way ANOVA with Tukey post-hoc test for multiple comparisons. *p < 0.05, **p < 0.01, and ***p < 0.001, ns, not significant change.
